# Beliefs about back pain and pain management behaviours, and their associations in the general population: A systematic review

**DOI:** 10.1002/ejp.1285

**Published:** 2018-08-07

**Authors:** L. Morton, M. de Bruin, M. Krajewska, D. Whibley, G.J. Macfarlane

**Affiliations:** ^1^ Epidemiology Group University of Aberdeen UK; ^2^ Aberdeen Centre for Arthritis and Musculoskeletal Health University of Aberdeen UK; ^3^ Health Psychology Group University of Aberdeen UK; ^4^ Arthritis Research UK/MRC Centre for Musculoskeletal Health and Work Southampton UK; ^5^ Department of Medical Informatics, Biometry and Epidemiology Ludwig Maximilian University of Munich Germany

## Abstract

Previous mass media campaigns have aimed to influence how people manage back pain, with mixed success. Campaigns should target beliefs which are related to the behaviours they aim to change. This systematic review brings together research that has measured the prevalence of beliefs about back pain in the general population and factors associated with these beliefs, including future pain‐related outcomes. Five databases were searched up until April 2017. Quantitative studies which reported a measure of agreement with a belief about back pain, cross‐sectional associations, or associations between beliefs and future outcomes were eligible. Eligibility was assessed and data extracted independently by two authors. Results were tabulated and narratively synthesized. Nineteen studies from 10 countries were eligible (median study *n* [IQR] = 990.5 [524.75–2387.5]). Beliefs were measured using eight questionnaires and 57 stand‐alone items. Beliefs about back pain's negative consequences were common across countries and populations, whereas most samples did not hold fear‐avoidance beliefs. Beliefs about back pain's consequences were associated with pain and disability, but only one study investigated this specific relationship prospectively. No studies investigated whether beliefs are associated with future pain management behaviours. Agreement with certain beliefs (e.g. about negative consequences) was associated with sociodemographic characteristics (e.g. older age) and poorer self‐rated health. Interventions may benefit from targeting beliefs about the perceived negative consequences of back pain in these populations. However, future research should explore how beliefs prospectively influence the management of back pain.

**Significance:**

This review brings together studies which have assessed the prevalence of beliefs about back pain, and factors associated with holding them. It highlights that whether or not these beliefs represent important determinants of how people manage pain remains unknown.

## 1 Introduction

Current advice for people with back pain stresses the importance of self‐management, including remaining active, within the early stages of an episode and once pain becomes persistent (National Institute for Health and Care Excellence, 2017). In contrast, previous management advice included provision of bed rest (Deyo and Weinstein, 2001). To coincide with this shift in management approach, experts recognized that the public may need updated information about how to manage back pain (Buchbinder et al., 2001b). Four mass media campaigns which aimed to change perceived societal‐level beliefs about back pain and its management were therefore carried out in Australia, Norway, Canada and Scotland (Buchbinder et al., 2001b; Waddell et al., 2007; Werner et al., 2008; Gross et al., 2010). These campaigns are now generally viewed as having had mixed success, with apparent shifts in beliefs not necessarily translating into changes in measured outcomes, which were largely related to healthcare utilization and work absenteeism (Gross et al., 2012).

The strategy of these campaigns was to change individuals’ beliefs which may result in reducing healthcare utilization and work absenteeism related to back pain. When pooled across a population, even small changes in beliefs may result in substantial savings in these areas if certain beliefs about back pain are indeed important determinants of targeted behaviours (Buchbinder, 2008). Interventions aiming to change beliefs should be based on increasing endorsement of beliefs with existing low endorsement rates, replacing unhelpful beliefs and/or introducing new helpful beliefs (Hornik and Woolf, 1999; Hornik and Yanovitzky, 2003). It is also essential that the beliefs targeted are related to and influence the outcome of interest (Hornik and Yanovitzky, 2003; Fishbein and Cappella, 2006). Additionally, specific beliefs may be associated with specific personal characteristics, previous experiences of illness or culturally available information (Leventhal et al., 2016). Therefore, an understanding of whether specific characteristics are associated with specific beliefs could inform whether messages should be tailored and targeted for specific groups (Hawkins et al., 2008).

Beliefs about back pain (e.g. beliefs about the aetiology of pain, fear of pain or re‐injury and self‐efficacy beliefs) are thought to influence the interpretation of nociceptive signals, the development of (chronic) disability, and adjustment to pain (Main et al., 2010). Beliefs about back pain have also been described as factors which ‘may influence self‐management behaviours’ and which may ‘[suggest] better ability to cope with low back pain’ (Buchbinder et al., 2001b; Briggs et al., 2010). However, despite extensive research on beliefs about back pain, a synthesis of the literature looking at their prevalence, and factors associated with them (including management behaviours) has not been conducted. A systematic review of observational studies conducted within the general population was therefore carried out to answer the following questions:

- Which beliefs about back pain have been assessed? What is the prevalence of these beliefs?
- What factors are associated with the different beliefs that individuals hold about back pain?
- Are particular beliefs associated with, and do they predict, back pain management behaviours and pain‐related outcomes?

## 2 Methods

This systematic review adhered to the PRISMA checklist for the reporting of systematic reviews. Its protocol was registered on PROSPERO (registration number: CRD42016038374).

### 2.1 Inclusion and exclusion criteria

Inclusion and exclusion criteria are listed in Table 1 which, taken together, identified observational studies where a quantitative measure of a belief about back pain was measured within a sample drawn from the general population. Baseline measures from trials or intervention studies were only included if their sample was drawn from a general population‐based sampling frame. Studies were excluded if they did not report on unique data from an original research study or if observational measures were collected during an explicitly described, concurrent intervention. Additionally, studies were excluded from the second and third research questions if they did not report the strength and direction of observational associations.

**Table 1:** Inclusion and exclusion criteria

**An article was included if it:** Reported a quantitative measure of a belief about back pain; ○ Beliefs about pain which were not specifically about back pain were also included if individuals reported concurrent back pain and if a belief measure asked about ‘their pain’. ○ Articles reporting on the prevalence of a belief had to report its prevalence within the entire sample relevant to the article and not within purposively selected subgroups. ○ Articles reporting a cross‐sectional or longitudinal relationship between a belief and some other factor were included if they reported the strength or direction of the relationship. Reported on a sample drawn from a general population sampling frame; Was published in a peer‐reviewed journal
**An article was excluded if it:** Did not contribute unique data; Did not measure or report beliefs about back pain in the absence of an explicit intervention for back pain; Was one of the following types of publication: editorial, commentary, meeting abstract, dissertation, unpublished manuscript, book, book chapter, guideline, (systematic) review; For cross‐sectional and prospective associations, did not report the strength or direction of the relationship between a belief about back pain and another factor or only reported group‐based differences

Cross‐sectional associations where a belief about back pain was specified by the original paper as the dependent variable, or as a correlate of a sociodemographic or general health‐related factor, were included for the second research question. This allowed for an understanding of whether different factors may be associated with being more likely to hold a given belief – if so, future research and intervention development may ultimately benefit from testing tailored or targeted materials based on these identified groups or characteristics.

Cross‐sectional and longitudinal studies were included to address the third research question. Specifically, associations where a belief about back pain was specified by the original paper as the independent variable were included. The inclusion of cross‐sectional and longitudinal study designs within the third research question allowed for a thorough synthesis of factors which may be important outcomes of holding given beliefs.

It is not possible to disentangle the temporality of relationships between variables within cross‐sectional studies – however, separating findings based on whether a given belief about back pain was specified as a dependent (research question 2) or independent (research question 3) variable allowed for a transparent report of the hypotheses underpinning the original studies. The synthesis of these studies provides a comprehensive map of how factors which are potentially associated with beliefs about back pain have been investigated in the literature to date.

### 2.2 Search methods

A systematic search was conducted using MEDLINE, PsycINFO, Embase, ISI Web of Science and CINAHL databases up until 21 April 2017. The search consisted of both text and medical subject heading (MeSH) terms for ‘back pain’, ‘beliefs’ and ‘general population’ which were generated using terms identified from the literature as well as others which were deemed important to include in order to best capture research relevant to the aims of the review. Full search details are provided in Supporting Information Appendix S1. No restrictions were applied to the searches. Reference lists of included articles were checked for any eligible studies not detected by the search.

### 2.3 Screening

Titles and abstracts were screened prior to assessing full‐text articles for eligibility. A random selection of 20% of titles and abstracts were dual‐screened and the high inter‐coder reliabilities suggested single‐coder screening was appropriate. All full‐text articles were assessed against eligibility criteria by two independent reviewers.

### 2.4 Data extraction and quality assessment

Two reviewers independently extracted data on study design and characteristics, sample characteristics, the back pain belief measure used and its prevalence and/or relationships with other factors, analysis methods and results. This data was extracted using an electronic form which was piloted prior to formal data extraction.

The quality of included articles was assessed using a modified version of the Quality in Prognostic Studies (QUIPS) tool which can be used to assess risk of bias in both cross‐sectional and longitudinal observational studies (Hayden et al., 2013; Mansfield et al., 2016). Articles were assessed, where applicable, on the following domains, each of which were assessed with multiple constituent items: ‘study participation’ (e.g. adequate description of the sampling frame and recruitment), ‘study attrition’ (e.g. description of attempt(s) to contact those lost to follow‐up), ‘belief’ and ‘outcome measurement’ (e.g. validity and reliability of measures used), ‘confounding’ (e.g. measurement of potentially important confounders) and ‘statistical analysis/reporting’ (e.g. adequate presentation of the data to assess the analytic strategy). Within each domain, a study could receive a qualitative rating of ‘low’, ‘moderate’ or ‘high’ risk of bias. To determine this overall rating for a domain, the ratings of the underpinning constituent items were assessed and an overall judgement was made. Where the risk of bias for more than one of the constituent items for a given domain was assessed as ‘unclear’, an article received an overall assessment of ‘unclear’ for that domain. The creators of the QUIPS tool advise against using a summative quality assessment score (Hayden et al., 2013); quality was therefore assessed and considered within each domain. Studies were not excluded based on the outcome of the quality assessment process. The findings presented in the results sections are qualified in terms of the quality of evidence which informs them.

Two reviewers independently extracted data and conducted quality assessment for all eligible articles. Any differences between reviewers were identified and discussed until consensus was reached.

### 2.5 Data analysis

The belief measures that were used were tabulated to illustrate the variety of the belief constructs that have been assessed within the general population. In the case of unique belief items, a thematic analysis was conducted independently by two reviewers to aid synthesis of results across qualitatively similar but distinct verbatim items. Disagreements regarding thematic analysis were resolved through discussion.

It was expected that articles describing the prevalence of different beliefs about back pain would report either (1) the proportion of the sample who agreed with a particular belief, represented by a simple percentage, or (2) a measure of central tendency if a questionnaire instrument was used. In the case of the latter, scores were interpreted within the context of a questionnaire's ‘neutral’ score (i.e. ‘neither agree nor disagree’) to ascertain whether a sample agreed or disagreed, on average. Calculation of the ‘neutral’ score was based on the Likert scale used and the number of items within the questionnaire. If 95% confidence intervals were not reported they were calculated using available data. Cross‐sectional or prospective relationships between beliefs about back pain and other factors were tabulated and narratively synthesized. Factors which were assessed as possibly being associated with specific beliefs about back pain were measured in different ways between studies which meant a quantitative synthesis using meta‐analytic approaches was not possible. Due to the observational nature of the studies included in the review, the terms, ‘predictor’ and ‘outcome’ are used within the synthesis of longitudinal studies (research question 3) as statistical terms. When multiple publications reported on the same measure or relationship within the same cohort, the publication with the largest sample size was included.

## 3 Results

### 3.1 Study selection

**Figure 1:**
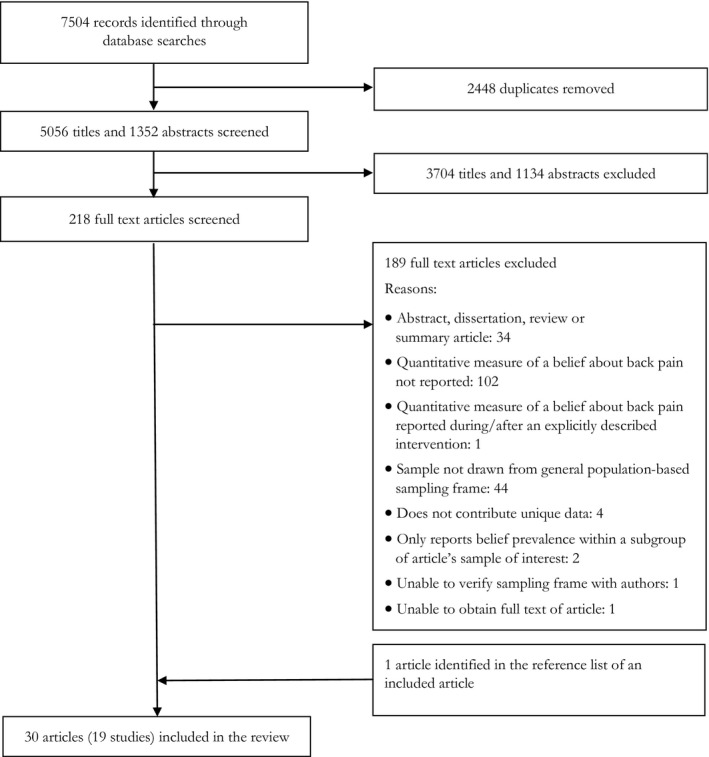
Identification and selection of included articles.

The searches returned 5056 unique results of which 30 articles met eligibility criteria. Details of the selection process are shown in Fig. 1. Dual title screening resulted in 93% agreement (Cohen's kappa = 0.83), dual abstract screening in 96% agreement (Cohen's kappa = 0.82) and full‐text screening resulted in 96% agreement (Cohen's kappa = 0.83).

### 3.2 Study characteristics

The 30 eligible articles represented analyses of data collected by 19 studies carried out across 10 countries in Europe (*n *= 12), Australasia (*n *=* *6) and North America (*n *=* *1). Within the studies whose total number of unique participants we could be certain of, sample size ranged from 54 to 5360 (median [IQR] = 990.5 [524.75–2387.5]). Publication years ranged from 1989 to 2015, with most articles published within the last decade (53%). The majority (57%) reported on individuals regardless of their personal experience of back pain; the others reported on individuals with or without experience of back pain at the present time/within the past month/6 months/year/ever. Details of the included articles are provided in Supporting Information Appendix S2.

### 3.3 Quality assessment

Thirteen articles were rated as being at moderate risk of bias and one was rated high risk of bias for ‘study participation’, due predominantly to inadequate descriptions of the sampling frame, the period and place of recruitment, and low to medium response rates. We were unable to ascertain the level of risk associated with five articles’ belief measurement due to uncertainty around the reliability and validity of belief measurement items and poor reporting on whether an adequate proportion of the sample had complete data. Twenty‐four of the remaining articles were at low risk of bias within this domain. Seven articles were at moderate risk of bias for ‘study confounding’ due mostly to neither specifying the reliability or validity of these measures nor accounting for potentially important confounders in a given relationship (e.g. experience of back pain). Details of the quality assessment for each study are presented in Table 2.

**Table 2:** Risk of bias of included studies: quality assessment using the QUIPS tool

Overarching project & associated studies (first author, year)	Quality assessment domains					
	Study participation	Study attrition	Belief measurement	Prospective outcome measurement	Consideration of confounders	Statistical analysis & reporting
Australia Back Pain Campaign and follow‐up						
Buchbinder (2001a)	Moderate	‐	Low	‐	‐	‐
Buchbinder and Jolley, (2005)	Low	‐	Low	‐	‐	‐
Dutch Population‐based Musculoskeletal Complaints and Consequences Cohort (DMC Cohort)						
Houben (2005), Leeuw (2007), Picavet (2002)^a^	Low	Moderate^a^	Low	Low^a^	Low	Low
Middle Sweden Back Pain Project						
Buer (2002), Linton (2000a)^a^, Linton (2000b)	Low	Low^a^	Low	Low^a^	Moderate	Low
Norway Back Pain Campaign						
Werner (2008)^a^, Werner (2009)	Moderate	‐	Unclear	‐	Low^a^	Low^a^
Norway Monthly Omnibus Survey						
Ihlebæk (2003)^a^, Ihlebæk (2005)	Moderate	‐	Low	‐	Moderate^a^	Low^a^
Södermanland Back Pain Project						
Linton (2001); Linton (2005)^a^	Moderate	High^a^	Low	Low^a^	Low^a^	Low^a^
Switzerland Musculoskeletal Health Survey						
Elfering (2009)^a^, Elfering (2015)^a^, Mannion (2009), Mannion (2013)	Moderate	Moderate^a^	Low	Low^a^	Low	Low
Standalone Projects/Publications						
Beales (2015)	Low	‐	Low	‐	Low	Low
Bowey‐Morris (2011)	Low	‐	Low	‐	Low	Low
Briggs (2010)	Moderate	‐	Low	‐	Moderate	Moderate
Darlow (2014)	Low	‐	Low	‐	‐	‐
Gross (2010)	Moderate	‐	Low	‐	‐	‐
Kovacs (2011)	Low	‐	Low	‐	‐	‐
Lindal (1989)	Low	‐	Moderate	‐	‐	‐
Szpalski (1995)	Low	‐	Unclear	‐	Moderate	Low
Urquhart (2008)	Low	‐	Low	‐	Low	Low
Vidal (2013)	Low	‐	Low	‐	‐	‐
Waddell (2007)	High	‐	Unclear	‐	‐	‐
Walker (2003)	Low	‐	Unclear	‐	Low	Low

a Indicates a quality assessment domain which is only of relevance to a specified publication(s) within an overarching study.

### 3.4 Belief measures and their constructs

Eight different questionnaire instruments were used to assess individuals’ beliefs about back pain. They included measures of beliefs about back pain's consequences, fear‐avoidance beliefs and measures of catastrophizing. Details of each instrument are provided in Supporting Information Appendix S3.

In addition to these eight questionnaire instruments, 57 stand‐alone belief statements were used in 10 studies. These statements represented beliefs about (1) the consequences of back pain, (2) the risks or benefits of activity while experiencing back pain, (3) the importance of rest during an episode of back pain, (4) the role of medicine in treating back pain, (5) the necessity of medical care or treatment, (6) diagnostic imaging or receiving a diagnosis for back pain, (7) causal attributions (e.g. heavy lifting), (8) prognosis and back pain's natural history, (9) psychological influences on recovery, (10) understanding the back in terms of its vulnerability, (11) understanding the relationship between pain and injury, and (12) understanding what back pain is like. A table of all stand‐alone items is provided in Supporting Information Appendix S4. The number of studies using each of these questionnaire instruments or stand‐alone belief items is described in Supporting Information Appendix S5.

### 3.5 Prevalence of specific beliefs about back pain in the general population

Sixteen studies contributed to estimating the prevalence of specific beliefs about back pain.

#### 3.5.1 Beliefs about back pain's consequences – Back Beliefs Questionnaire

**Figure 2:**
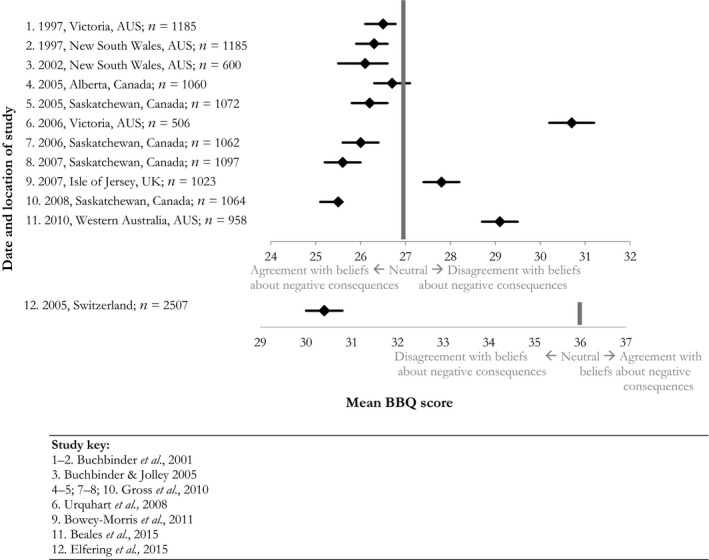
Beliefs about back pain's negative consequences – Back Beliefs Questionnaire mean scores with 95% Confidence Intervals.

Beliefs about the inevitability of negative consequences resulting from an episode of back pain, such as having periods of time off work and ending up in a wheelchair, were measured using the Back Beliefs Questionnaire (BBQ) in 12 samples who represented mixed individuals with regard to personal experience of back pain (median *n* [IQR] = 1063 [1006.75–1119]). Fig. 2 shows a trend where agreement with beliefs about these consequences was reported within eight samples, whereas weak to strong disagreement with beliefs about back pain's consequences was reported within four samples. There were no obvious differences in terms of study quality or design between these studies, but one of the four which disagreed with these beliefs was a sample of adults aged 46–64 (Beales et al., 2015) and another was an all‐female sample (Urquhart et al., 2008). The latter took place in Victoria, Australia which was also the location of one of the previously described back pain mass media campaigns (Buchbinder et al., 2001b). Data collection for this study started 7 years after the active period of the mass media campaign (Buchbinder et al., 2001b; Urquhart et al., 2008). All other studies represented males and female adults from broad age ranges. Overall, eight out of 12 samples agreed on average with beliefs that back pain has inevitable negative consequences (Fig. 2).

#### 3.5.2 Beliefs about back pain‐related fear and activity avoidance – Fear‐Avoidance Questionnaires

**Figure 3:**
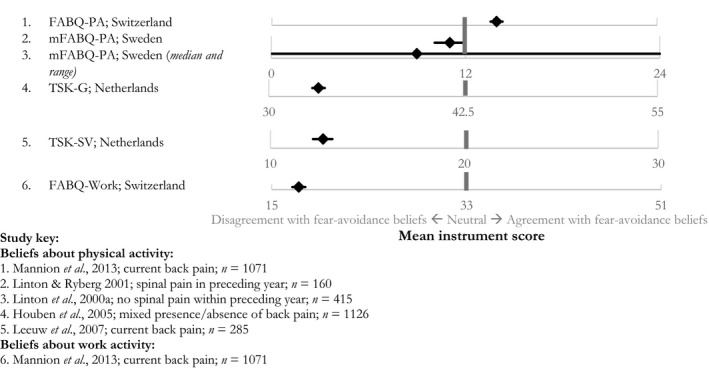
Fear‐avoidance beliefs: mean questionnaire scores with 95% Confidence Intervals (unless otherwise specified).

Agreement with beliefs about fearing and avoiding physical activity was reported within four studies. Most samples (median *n* [IQR] = 415 [285–1071]) represented individuals with current or recent experience of back pain. There was substantial heterogeneity in endorsement rates, with two studies strongly disagreeing and three reporting just below and above neutral scores (Fig. 3). The single study which investigated fear‐avoidance beliefs about work‐related activities reported disagreement on average with these beliefs (Fig. 3). Of these studies, Picavet et al. (2002) also reported the proportion of their sample who agreed with six of the 17 items on the Tampa Scale for Kinesiophobia‐General Population. The proportion of agreement ranged substantially across items. For example, only 12% (95% CI: 10.7–13.9) thought that is was ‘really not safe’ for someone with low back pain to be physically active while 73% (95% CI: 70.8–75.2) thought that there would not be ‘much back pain if there weren't something wrong with the back’.

#### 3.5.3 Stand‐alone belief statement items

**Figure 4:**
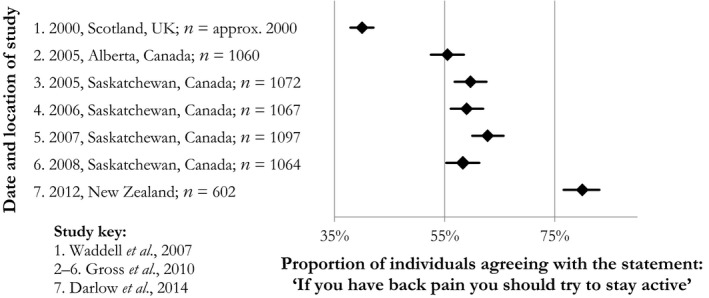
Prevalence of agreement with the belief: ‘If you have back pain you should try to stay active’.

We identified 57 stand‐alone belief items, and the proportion of agreement with only three was reported across more than one study (Supporting Information Appendix S4). These three items represented beliefs about activity or rest. The percentage agreement with a given belief varied substantially between countries and study year. For example, a belief about trying to stay active when in pain was endorsed by 40% (95% CI: 37.9–42.1) of adults in Scotland in 2000 versus 80% (76.6–83.1) in New Zealand in 2012 (Fig. 4) (Waddell et al., 2007; Gross et al., 2010; Darlow et al., 2014). The prevalence of agreement with a belief about the need to take it easy/rest until pain improves also varied across several studies, with 25 to 70% of samples endorsing this belief, depending on the study which varied by country and year of data collection (Ihlebæk and Eriksen, 2003, 2005; Waddell et al., 2007; Werner et al., 2008; Gross et al., 2010; Darlow et al., 2014).

### 3.6 Sociodemographic, health‐ and pain‐related factors associated with beliefs about back pain: results from cross‐sectional studies where beliefs were hypothesized to represent the dependent variable or correlate of a sociodemograpic/general health variable

Five studies reported a cross‐sectional analysis linking back pain beliefs to sociodemographic, general health and back pain‐related factors within samples who were mixed with regard to their experience of back pain (Supporting Information Appendix S6, sample *n* median [IQR] = 1023 [1015–1071]). Most relationships were only investigated within single studies and most were rated as having low risk of bias across the quality assessment domains, however three were at moderate risk of bias within the ‘study participation’ domain due predominantly to inadequate descriptions of the source population and sampling frame and/or moderate response rates (Ihlebæk and Eriksen, 2003; Werner et al., 2008; Elfering et al., 2009; Mannion et al., 2013). Sociodemographic factors which were consistently (i.e. within >1 study) associated with more agreement with beliefs about the negative consequences of back pain included: being older (two studies; Mannion et al., 2013; Beales et al., 2015), having completed less education (two studies; Bowey‐Morris et al., 2011; Mannion et al., 2013) and having a lower income (two studies; Mannion et al., 2013; Beales et al., 2015). General health‐related factors which were consistently associated with agreement on these beliefs included having poorer self‐rated general health (two studies; Bowey‐Morris et al., 2011; Mannion et al., 2013) and poorer mental well‐being or mental health (two studies; Mannion et al., 2013; Beales et al., 2015). Back pain‐related factors which were investigated as hypothesized explanatory factors of one's beliefs about back pain were only assessed within single studies but included: reporting more limitations in activities of daily living (Bowey‐Morris et al., 2011), accepting back pain‐related work absence (Bowey‐Morris et al., 2011), having previously missed work due to back pain (Bowey‐Morris et al., 2011), being on sick leave/disability (Beales et al., 2015) and having had more recent experience of back pain (Beales et al., 2015). Three other studies reported correlations between beliefs and back pain‐related variables but did not specify nor test the direction of the relationship (Linton et al., 2000a; Briggs et al., 2010; Mannion et al., 2013; Elfering et al., 2015; Supporting Information Appendix S7).

### 3.7 Hypothesized outcomes of beliefs about back pain: results from cross‐sectional and longitudinal studies where beliefs were specified as the independent variable

Eight studies (median sample *n* [IQR] = 720 [486–1228]) reported on whether one's beliefs about back pain were associated with pain, disability, work and illness‐behaviour outcomes (Table 3). Four of these reported on prospective relationships, and of these, three were at moderate to high risk of ‘attrition bias’ due predominantly to not providing a description of attempts to contact participants who were lost to follow‐up, and/or providing an inadequate description of those lost to follow‐up and whether there were important differences between these individuals and the initial sample (Picavet et al., 2002; Linton, 2005; Elfering et al., 2009, 2015). Two were at risk of ‘participation bias’ (e.g. inadequate descriptions of sampling frame/source population, period/place of recruitment; Elfering et al., 2009, 2015; Linton, 2005). The cross‐sectional evidence (8 studies) was generally of high quality with the exception of two studies which were at moderate risk of ‘participation bias’ (Linton, 2005; Mannion et al., 2009, 2013; Elfering et al., 2015) and two which did not measure and/or take into account potentially important confounders (Szpalski et al., 1995; Linton et al., 2000b). With the exception of two studies, all investigated relationships where the belief of interest was about back pain's consequences or fear‐avoidance beliefs.

**Table 3:** Factors associated with beliefs about back pain: results from cross‐sectional and longitudinal studies where beliefs about back pain were hypothesized to represent the independent variable

Article	Sample (country, *n*, description)	Belief	Dependent variable	Statistical analysis	Results
**Cross‐sectional studies**					
Elfering et al. (2015)	Switzerland, 2507, mixed BP	BBQ	Pain intensity (0–6 scale)	Pearson correlation, cross‐lagged panel regression analysis	Pain intensity associated with BBQ scores in those with BP (β = 0.38, *p *< 0.001) and in overall sample (β = 0.17, *p *< 0.001)
Linton et al. (2000b)	Sweden, 720, mixed BP	mFABQ‐PA	Membership of moderate pain intensity group (pain ≥5 on 0–10 scale, <30 days sick leave, not on disability pension)	Discriminant analysis	mFABQ‐PA scores associated with moderate pain intensity at first stage of discriminant analysis (*p *< 0.05) but did not contribute to the final combined model
Linton (2005)	Sweden, 521, mixed BP	mFABQ‐PA	Significant pain intensity (pain >6 on 0–10 scale)	Univariate logistic and multiple regression	Significant pain associated with mFABQ‐PA within univariate (OR: 1.72, 95% CI: 1.21–2.46, *p *< 0.01) but not multiple regression analyses
Urquhart et al. (2008)	Australia, 506, mixed BP, female	BBQ	Pain intensity (tertiles based on 0–100 pain rating scores); disability (Chronic Pain Grade)	Univariate and multiple ordinal regression	High pain intensity and worse disability associated with BBQ scores in multiple regression analyses (OR: 0.93, 95% CI: 0.88–0.99, *p *<* *0.05; OR: 0.93, 95% CI: 0.89–0.97, *p *<* *0.001)
Leeuw et al. (2007)	Netherlands, 152, current BP (subset of Houben et al., 2013)	TSK‐SV	Disability (Quebec BP Disability Scale)	Pearson correlation, stepwise regression analysis	Disability associated with TSK‐SV after accounting for age, gender, chronicity, catastrophising, pain intensity and baseline disability (β = 0.20, *p *<* *0.01)
Buer and Linton (2002)	Sweden, 917, mixed BP (overlaps with Linton, 2000b sample)	mFABQ‐PA	Pain intensity (no, mild, moderate pain); limitations in activities of daily living (within people with BP)	Logistic regression adjusted for catastrophising, smoking, gender	mFABQ‐PA associated with limitations in activities of daily living, but not pain intensity (*p *<* *0.05)
Houben et al. (2005)	Netherlands, 1126, mixed BP	TSK‐G	SF‐36: Physical Functioning, Social Functioning, Role Restrictions, Pain, General Health subscales; Pain Catastrophising Scale	Pearson correlation, univariate regression adjusted for gender, age, back pain status	All SF‐36 subscales and pain catastrophising associated with TSK‐G scores (β range for different subscales=−0.27 to 0.21, *p *<* *0.001; β = 0.38, *p *<* *0.001, respectively)
Beales et al. (2015)	Australia, 486, BP within preceding month, age ≥49	BBQ	Disability (Oswestry Disability Index); ‘interferes with normal activities’; ‘interferes with physical activities’; ‘usually causes work absence’; ‘usually use medication for BP’; ‘usually seek professional care for BP’	Univariate logistic regression analysis and adjusted analyses for current pain level, age, SF‐12, income, employment.	Disability, interference in normal and physical activities, work absence and medication use associated with BBQ in univariate analyses. Only disability and interference in normal activities associated in adjusted analyses (B: −0.37, 95% CI: −0.49 to 0.25, *p *<* *0.001; OR: 0.95, 95% CI: 0.92–0.98, *p *<* *0.01, respectively)
Mannion et al. (2009)	Switzerland, 670, current BP and employed (subset of Elfering et al., 2015)	BBQ; FABQ‐PA and Work	Perceived reduction in work productivity; *n* days missed work	Spearman rank correlation, hierarchical multiple regression	All beliefs correlated with perceived reduction in work productivity and *n* days absent. Only FABQ‐Work associated with both outcomes within multiple regression (β = 0.26, *p *<* *0.001; β = 0.15, *p *<* *0.001 respectively)
Mannion et al. (2013)	Switzerland, 1071, current BP (overlaps with Elfering et al., 2015)	BBQ; FABQ‐PA and Work	Sought healthcare for BP within preceding 4 weeks	Pearson correlation, univariate and multiple logistic regression	All beliefs associated with healthcare‐seeking within univariate analysis. Only FABQ‐Work associated within multiple regression (OR: 1.025, 95% CI: 1.005–1.044, *p *<* *0.05)
Szpalski et al. (1995)	Belgium, 2660, history of BP	Statement about whether BP would be lifelong problem.	‘Sought healthcare’; ‘had bed rest’; ‘took medicine’; ‘had X‐ray’; ‘had surgery’	Multiple logistic regression	All previous illness behaviours except having had surgery were associated with agreement with belief after accounting for sociodemographic factors and daily BP (*p *<* *0.001)
Walker et al. (2004)	Australia, 1228, BP within preceding 6 months	Statement about fear that BP would impair future work capacity.	Sought healthcare in preceding 6 months	Univariate logistic regression	Agreement with belief associated with having sought healthcare (OR: 2.2, 95% CI: 1.7–2.9)
**Longitudinal studies**					
Linton et al. (2000a)	Sweden, 449, no spinal pain within previous 12 months	mFABQ‐PA	At 12 months: occurrence of pain and test of physical functioning	Logistic regression analyses adjusted for BMI, age, gender, smoking	Above‐median scores on mFABQ‐PA predicted future occurrence of pain (OR: 2.04, 95% CI: 1.19–3.48) and physical functioning (OR: 1.70, 95% CI: 1.12–2.59)
Picavet et al. (2002)	Netherlands, 1571, either presence (*n *=* *411) or absence (*n *=* *1160) of BP at baseline	TSK‐G; PCS	At 6 months: presence of BP, BP with limitation in daily activities, BP which has lasted ≥3 months, severe BP (≥5 on 1–10 scale), BP with disability (Quebec BP Disability Questionnaire)	Logistic regression analyses adjusted for baseline pain severity and disability in analysis for those with BP at baseline	Within people without BP at baseline, TSK‐G predicted disabling BP (OR: 3.1, 95% CI: 1.1–8.7) but no other outcomes at follow‐up. Within people who had BP at baseline, TSK‐G and PCS predicted BP at follow‐up (unadjusted), and severe pain, chronic pain, and pain with limitation in daily activity (adjusted). TSK‐G, but not PCS, predicted disabling pain (adjusted)
Linton (2005)	Sweden, 372, absence of BP at baseline	mFABQ‐PA	At 6 months: Significant pain intensity (pain >6 on 0–10 scale)	Logistic regression adjusted for catastrophising	mFABQ‐PA did not predict occurrence of future significant pain (OR: 1.48, 95% CI: 0.66–3.32)
Elfering et al. (2015)	Netherlands, 1833, mixed BP	BBQ	At 12 months: Pain intensity (0–6 scale)	Cross‐lagged structural equation panel modelling	BBQ predicted future pain intensity in the overall sample (β = 0.6, *p *<* *0.05), in those who had BP (β = 0.11, *p *<* *0.05), who did not exercise (β = 0.15, *p *<* *0.05), and who had BP and did not exercise at baseline (β = 0.29, *p *<* *0.05), but not in those who did not have BP, who exercised, and who had BP and exercised at baseline
Elfering et al. (2009)	Netherlands, 264, presence of BP at baseline	BBQ; FABQ‐PA and Work	Weekly pain intensity (0–6 scale), pain frequency, recovery, work impairment	Multi‐level model	Pain intensity predicted by FABQ‐PA and Work; pain frequency predicted by FABQ‐Work; recovery and perceived work impairment predicted by BBQ and FABQ‐Work

Cross‐sectional evidence demonstrated that more agreement with beliefs about back pain's consequences was associated with higher pain intensity (two studies; Urquhart et al., 2008; Elfering et al., 2015), disability (two studies; Urquhart et al., 2008; Beales et al., 2015), recent healthcare‐seeking for back pain (two studies; Walker et al., 2004; Mannion et al., 2013), previous medication use (one study; Beales et al., 2015), and previous back pain‐related work absence (one study; Beales et al., 2015). Prospectively, more agreement with beliefs about back pain's consequences predicted higher pain intensity at 12 months (Elfering et al., 2015), and weekly perceived work impairment and reduced recovery over the course of 1 year within one study (Elfering et al., 2009).

Similarly, cross‐sectional evidence illustrated that more agreement with fear‐avoidance beliefs was associated with the presence or intensity of one's pain (two studies; Linton et al., 2000b; Linton, 2005), disability (two studies; Buer and Linton, 2002; Leeuw et al., 2007), recent healthcare‐seeking (one study; Mannion et al., 2013), general health measures and catastrophizing (one study; Houben et al., 2005). Fear‐avoidance beliefs about work‐related activities were associated with recent healthcare‐seeking (one study; Mannion et al., 2013), recent back pain‐related work absence (one study; Mannion et al., 2009) and a perceived reduction in work productivity (one study; Mannion et al., 2009). Prospectively, more agreement with fear‐avoidance beliefs predicted future presence or intensity of pain (three studies; Linton et al., 2000a; Picavet et al., 2002; Elfering et al., 2009) and disability (two studies; Linton et al., 2000a; Picavet et al., 2002) at 6 and 12 months; fear‐avoidance beliefs about work‐related activities also predicted weekly perceived reductions in work productivity, higher frequency of pain episodes and reduced recovery (one study; Elfering et al., 2009).

Within a cross‐sectional analysis, holding a belief about whether back pain was perceived to be a lifelong problem was associated with previous healthcare‐seeking, bed rest, medicine use, X‐ray and surgery for back pain (one study; Szpalski et al., 1995). Prospectively, back pain‐related catastrophizing predicted future pain intensity and disability at 6 months (one study; Picavet et al., 2002).

## 4 Discussion

### 4.1 Principal findings

While the prevalence of a number of beliefs has been assessed within the general population, presumably because they are thought to be important for back pain‐related outcomes, comparatively little research has investigated these associations prospectively. Beliefs about the inevitability of negative consequences that might come from an episode of back pain were common across countries and populations. We identified consistent evidence that these beliefs were associated with pain‐ and disability‐related measures as well as previous healthcare‐seeking for back pain – however, most observed relationships were assessed within cross‐sectional studies and did not always account for potentially important confounders. Beliefs about back pain's negative consequences were consistently associated with being older, having completed less education, having a lower income and having poorer self‐rated general and mental health; interventions may therefore benefit from targeting beliefs about the perceived negative consequences of back pain within these populations, if future research clarifies the role that these beliefs may have for pain‐related outcomes including management behaviours.

### 4.2 Comparison with existing literature

Due to the heterogeneity of measurement of beliefs about back pain in the general population, comparisons across different temporal and geographic contexts were problematic. However, people tended to hold beliefs about the negative consequences which might come from an episode of back pain but disagree with beliefs about fearing and avoiding physical activity. Possible associations between sociodemographic/health‐related factors and holding specific beliefs about back pain were not often investigated, but studies that did tended to explore links between sociodemographic factors and beliefs about back pain's negative consequences. Beyond these relationships, we cannot describe which sociodemographic/health‐related measures may be associated with other beliefs within the general population. Similarly, despite the breadth of research which has described different beliefs people have about back pain, beliefs about back pain's consequences and fear‐avoidance beliefs were those most commonly used as predictors of future back pain‐related outcomes, which were most often related to pain and disability. While there was some evidence for fear‐avoidance beliefs to be associated with future work impairment, this was only investigated within one study and no research investigated, prospectively, whether different beliefs may be associated with future back pain management behaviours. Previous mass media campaigns have aimed to influence individuals’ beliefs about back pain and management behaviour by providing reassurance that back pain is not often indicative of a serious condition, providing advice to remain active, and by trying to change expectations of healthcare services (Buchbinder et al., 2001b; Waddell et al., 2007; Werner et al., 2008; Werner and Gross, 2009; Gross et al., 2010). As previously described, most of campaigns are now viewed as not having been overly successful in achieving their ultimate aims to influence outcomes related to healthcare utilization and work absenteeism. Based on the current evidence highlighted in this review, it is unclear which beliefs are associated with and may therefore influence how people manage their pain in the future. Of the campaigns carried out to date, the Australian campaign is considered the most successful and so it is possible that it better attended to targeting beliefs which were particularly implicated in their measured outcomes. Within this campaign in particular, potentially important contextual factors were also considered (e.g. delivery of messages by well‐known celebrities, endorsement by clinical organizations) and incorporated into a multifaceted intervention which also operated within workplaces and healthcare practices (Buchbinder et al., 2001a,b). This consideration of intervention context may have served to bolster messages which ultimately led to the subsequent improvements in measured outcomes.

Various models of management and illness behaviour have recognized the importance of individual‐level factors, including an individual's perceptions of illness, in influencing management and healthcare‐seeking behaviours (Wyke et al., 2013). Most of the research identified within this review has instead focused on pain‐ and disability‐related measures associated with specific beliefs about back pain, which can be viewed within the context of the Fear‐Avoidance Model of Pain (Vlaeyen et al., 2016). This model hypothesizes that the experience of pain is interpreted as being of high or low threat, the former leading to fear of pain and subsequent avoidance of activities perceived to cause pain. It is this avoidance of activity which is hypothesized to reflect and contribute to the development of chronic pain and disability (Vlaeyen et al., 2016).

The evidence in this review highlighted that the beliefs of individuals who had current or recent experience of back pain were associated with their pain intensity and disability. This cross‐sectional evidence makes it impossible to disentangle which (if either) came first, but both pathways are plausible and both may be important (Linton and Shaw, 2011). A sensation of pain could influence an individual's interpretation of it which is then reflected by reporting more ‘negative’ beliefs about it. Indeed, individuals learn from their personal experiences of pain and this psychological process of learning from pain is important for survival (Linton and Shaw, 2011). Conversely, an individual who, over time, has developed a specific representation of back pain may be more likely to interpret and report their pain as being more or less severe. This latter hypothesis is illustrated by results from experimental studies which have demonstrated that verbal suggestions about what to expect from pain modulate the individual's experience of it (Peerdeman et al., 2016). These two avenues mirror research within the back pain literature – hypotheses have been put forward that individuals, based on different experiences, may be ‘learned’ or ‘misinformed’ activity avoiders – and these different groups may warrant different treatment strategies (Pincus et al., 2010).

To further elucidate this cross‐sectional evidence, prospective evidence within the review suggested that beliefs were associated with future back pain and disability outcomes but these relationships did not always account for important baseline variables. These associations indicated that individuals’ beliefs about back pain at one point in time may shape future experiences of it. Reflecting this pathway, *changes* in one's understanding of their back pain have also been shown to uniquely predict subsequent disability within a cohort of patients receiving acupuncture for their back pain, while, conversely, early changes in disability did not predict subsequent understanding of back pain (Bishop et al., 2015). A systematic review of reassurance within primary care settings on patient outcomes has also highlighted the importance that cognitive reassurance (an aspect of the consultation in which a healthcare practitioner specifically aims to change patients’ understanding of their illness through education), has on patients’ subsequent symptom improvement and healthcare utilization (Pincus et al., 2013).

### 4.3 Strengths and limitations

This review identified beliefs which have been measured in the general population as well as factors associated with holding these beliefs, including future outcomes. We are unable to comment on the beliefs of individuals within specific clinical or occupational contexts. However, the results of clinical studies, for example, would only be representative of individuals who have sought healthcare and the beliefs of these individuals may differ from those who have managed back pain within the community setting (e.g. Baird and Haslam, 2013; Sirri et al., 2013). Indeed, this was a motivating factor in our decision to focus on the assessment of beliefs in the general population. Additionally, our search strategy was focused on beliefs about back pain and its management rather than more general qualities or abilities like optimism and resilience. We feel it was robust in identifying all relevant studies with this focus, but we are unable to comment on the role that these qualities may also play in pain‐related outcomes (e.g. Conversano et al., 2010; Sturgeon and Zautra, 2010; Goubert and Trompetter, 2017).

The evidence included in this review is observational, therefore causality cannot be assumed. Prospective relationships did not always account for an individual's baseline level or history of pain, which could have not only affected their beliefs at that time (Bostick et al., 2013; Beales et al., 2015) but also their likelihood of developing pain or disability in the future. Because relationships between beliefs and management and illness behaviours have only been assessed cross‐sectionally for past behaviours, they cannot account for an individual's level of pain or disability at the time that they, for example, consulted a healthcare professional. Their reported belief could be a consequence, rather than a determinant, of management or illness behaviour. Indeed, qualitative work has highlighted the lasting role that interactions with healthcare providers may have on one's understanding of back pain (Darlow et al., 2013).

## 5 Conclusions

Despite a substantial amount of research which has investigated individuals’ beliefs about back pain, and cross‐sectional associations with other factors, comparatively little research has investigated relationships between these beliefs and future back pain‐related outcomes. Findings from single studies seemed to indicate that holding stronger fear‐avoidance beliefs and beliefs about back pain's consequences were each associated with future negative pain‐related outcomes. However, no research has investigated the prospective relationships between these beliefs and management behaviours. We recommend that future studies therefore also assess healthcare‐seeking and management behaviours in addition to pain‐related outcomes. This would allow for identifying the most important beliefs associated with those behaviours. This, in turn, is important for the future development of effective communication and education interventions which aim to change how people manage back pain within the general population setting.

## Author contributions

L. Morton: review inception, designed protocol, searched databases, identified eligible studies, extracted relevant data and conducted quality assessments, synthesized results, wrote first version of the current article. M. de Bruin: review inception, protocol development, interpretation of results. M. Krajewska: duplicate eligibility screening, duplicate data extraction and quality assessment. D. Whibley: duplicate eligibility screening and interpretation of results. G.J. Macfarlane: review inception, protocol development, interpretation of results. All authors critically reviewed earlier drafts of the article and contributed significantly to the final version. All authors discussed the results and commented on the manuscript.

## Supporting information


**Appendix S1** Details of search strategy: presented for MEDLINE but adapted for each database.
**Appendix S2** Description of empirical studies.
**Appendix S3** Description of questionnaire instruments measuring beliefs about back pain.
**Appendix S4** Characterisation of individual belief statements and prevalence of agreement with each statement (ranked within each thematic grouping according to prevalence of agreement).
**Appendix S5** Number of studies contributing to each review question using each of the described beliefs.
**Appendix S6** Factors associated with beliefs about back pain: results from cross‐sectional studies where beliefs were hypothesised as the dependent variable, or investigated as a correlate of a sociodemographic or general health‐related factor.
**Appendix S7** Correlates of beliefs about back pain: relationships between beliefs and factors other than sociodemographic/health variables (where the independent and dependent variables were not specified).
