# Supplementary material for: Beliefs about back pain and pain management behaviours, and their associations in the general population: A systematic review
Source: Eur J Pain. 2018 Aug 7;23(1):15–30. doi: 10.1002/ejp.1285 (PMC6492285; doi:10.1002/ejp.1285)
Supplement: Supplementary file 1 — Appendix S1 Details of search strategy: presented for MEDLINE but adapted for each database. Appendix S2 Description of empirical studies. Appendix S3 Description of questionnaire instruments measuring beliefs about back pain. Appendix S4 Characterisation of individual belief statements and prevalence of agreement with each statement (ranked within each thematic grouping according to prevalence of agreement). Appendix S5 Number of studies contributing to each review question using each of the described beliefs. Appendix S6 Factors associated with beliefs about back pain: results from cross‐sectional studies where beliefs were hypothesised as the dependent variable, or investigated as a correlate of a sociodemographic or general health‐related factor. Appendix S7 Correlates of beliefs about back pain: relationships between beliefs and factors other than sociodemographic/health variables (where the independent and dependent variables were not specified). [file EJP-23-15-s001.docx]

| Supporting Information Appendix S1. *Details of search strategy: presented for MEDLINE but adapted for each database* | |
| --- | --- |
| Back pain: | (all terms combined using the OR operator): exp Back Pain/ (MESH term); back pain.tw; low back pain.tw; backache.tw; back trouble.tw; lumbar pain.tw; spin* pain.tw |
| Beliefs: | (all terms combined using the OR operator): exp Attitude to Health/ (MESH term); exp Health Knowledge, Attitudes, Practice/ (MESH term); percept*.tw; belief*.tw; attitud*.tw; fear avoidan*.tw; fear.tw; self efficacy.tw; efficacy.tw; norm*.tw; expectation*.tw; locus of control.tw; myth*.tw; misconception*.tw; catastrophi*.tw; hypervigilan*.tw |
| General population: | (all terms combined using the OR operator): public*.tw; population*.tw; communit*.tw; random sampl*.tw |

| Supporting Information Appendix S2. *Description of empirical studies* | | | | | |
| --- | --- | --- | --- | --- | --- |
| First author, publication year, country, *n* (*relevant to review*) | Sampling frame and sample description with respect to BP experience. | Belief measure relevant to review | Contributions to research questions and specific associations investigated | Quality Assessment (*risk of bias*) | |
| Australia Back Pain Campaign (and follow-up) | | | | | |
| Buchbinder R, 2001a, Australia, 3549 | Individuals identified using random telephone number generation within two provinces.  Mixed BP. | BBQ | Prevalence (RQ1) | Participation: moderate  Attrition: n/a  Belief Measurement: low  Prospective Outcome Measurement: n/a  Study Confounding: n/a  Statistical Analysis & Reporting: n/a | |
| Buchbinder R, 2005, Australia, 600 | Individuals identified using random telephone number generation within two provinces.  Mixed BP. | BBQ | Prevalence (RQ1) | Participation: low  Attrition: n/a  Belief Measurement: low  Prospective Outcome Measurement: n/a  Study Confounding: n/a  Statistical Analysis & Reporting: n/a | |
| Dutch Population-Based Musculoskeletal Complaints and Consequences Cohort (DMC cohort) | | | | | |
| Houben RA, 2005, Netherlands, 1126 | Random sample of individuals aged ≥25 listed on 1998 Dutch population register, stratified by 10-year age groups and gender.  Mixed BP | TSK-G | Prevalence (RQ1);  TSK-G associations with SF-36 subscales and PCS (RQ3, cross-sectional) | Participation: low  Attrition: n/a  Belief Measurement: low  Prospective Outcome Measurement: n/a  Study Confounding: low  Statistical Analysis & Reporting: low | |
| Leeuw M, 2007, Netherlands, 152 | As described above in Houben *et al.*, 2005.  Presence of LBP at time of follow-up survey. | TSK-SV | Prevalence (RQ1);  TSK-SV association with Quebec BP Disability Scale (RQ3, cross-sectional) | Participation: low  Attrition: n/a  Belief Measurement: low  Prospective Outcome Measurement: n/a  Study Confounding: low  Statistical Analysis & Reporting: low | |
| Picavet HJ, 2002, Netherlands, 1571 | As described above in Houben *et al*., 2005.  Mixed BP. | TSK-G  PCS | Prevalence (RQ1);  TSK-G and PCS as predictors of future (limiting/ disabling/chronic/severe) BP (RQ3, prospective) | Participation: low  Attrition: moderate  Belief Measurement: low  Prospective Outcome Measurement: low  Study Confounding: low  Statistical Analysis & Reporting: low | |
| Middle Sweden Back Pain Project | | | | | |
| Buer N, 2002, Sweden, 917 | Random sample of individuals aged 35-45 from the population census registers of three communities in central Sweden.  In past year: No spinal pain; or spinal pain ≤4 (intensity) and sick-listed <15 days; or spinal pain ≥5 (intensity) and sick-listed <31. | mFABQ-PA | mFABQ-PA association with pain intensity (RQ3, cross-sectional) | Participation: low  Attrition: n/a  Belief Measurement: low  Prospective Outcome Measurement: n/a  Study Confounding: moderate  Statistical Analysis & Reporting: low | |
| Linton SJ, 2000a, Sweden, 449 | As described above in Buer *et al.*, 2002.  No spinal pain within past year. | mFABQ-PA | Prevalence (RQ1);  mFABQ-PA as predictor of future occurrence of BP and ability on a test of physical functioning (RQ3, prospective) | Participation: low  Attrition: low  Belief Measurement: low  Prospective Outcome Measurement: low  Study Confounding: moderate  Statistical Analysis & Reporting: low | |
| Linton SJ, 2000b, Sweden, 720 | As described above in Buer *et al.*, 2002.  In past year: no spinal pain; or ‘‘moderate” spinal pain (≥5 on 0-10 scale, >1 episode, <30 days sick leave). | mFABQ-PA | mFABQ-PA association with moderate pain intensity (RQ3, cross-sectional) | Participation: low  Attrition: n/a  Belief Measurement: low  Prospective Outcome Measurement: n/a  Study Confounding: moderate  Statistical Analysis & Reporting: low | |
| Norway Back Pain Campaign | | | | | |
| Werner EL, 2008, Norway, 2507 | Random sample of individuals from telephone databases of three Norwegian counties.  Mixed BP. | Study-specific items about: activity, rest, prognosis, diagnostic imaging, and medical treatment. | Prevalence (RQ1);  Beliefs’ association with county of residence (RQ2) | Participation: moderate  Attrition: n/a  Belief Measurement: unclear  Prospective Outcome Measurement: n/a  Study Confounding: low  Statistical Analysis & Reporting: low |  |
| Werner EL, 2009, Norway, 2507 | As described above in Werner *et al.,* 2008.  Mixed BP. | Study-specific items about diagnostic imaging. | Prevalence (RQ1) | Participation: moderate  Attrition: n/a  Belief Measurement: unclear  Prospective Outcome Measurement: n/a  Study Confounding: n/a  Statistical Analysis & Reporting: n/a | |
| Norway Monthly Omnibus Survey | | | | | |
| Ihlebæk C, 2003, Norway, 1015 | Random sample of adults from Norwegian telephone databases, proportionally stratified by municipality population.  Mixed BP. | Study-specific items about: rest, activity, medical treatment, diagnostic imaging, cause, understanding the experience of BP. | Prevalence (RQ1);  Beliefs’ association with age and level of completed education (RQ2) | Participation: moderate  Attrition: n/a  Belief Measurement: low  Prospective Outcome Measurement: n/a  Study Confounding: moderate  Statistical Analysis & Reporting: low | |
| Ihlebæk C, 2005, Norway, 1014 | As described above in Ihlebæk *et al*., 2003.  Mixed BP. | As described above in Ihlebæk *et al*., 2003. | Prevalence (RQ1) | Participation: moderate  Attrition: n/a  Belief Measurement: low  Prospective Outcome Measurement: n/a  Study Confounding: n/a  Statistical Analysis & Reporting: n/a |  |
| Södermanland Back Pain Project | | | | | |
| Linton SJ, 2001, Sweden, 175 | Random sample of individuals aged 35-45 from the population census registers of two communities in Sӧdermanland county, Sweden.  Within the past year: experience of spinal pain which was ≥7 on 0-10 scale at its worst and experienced ≥4 pain episodes. | mFABQ-PA | Prevalence (RQ1) | Participation: moderate  Attrition: n/a  Belief Measurement: low  Prospective Outcome Measurement: n/a  Study Confounding: n/a  Statistical Analysis & Reporting: n/a | |
| Linton SJ, 2005, Sweden, 581 | As described above in Linton *et al.*, 2001  Within the past year: no spinal pain; or ‘significant’ spinal pain (≥6 on 0-10 scale). | mFABQ-PA | mFABQ-PA as predictor of significant spinal pain (RQ3, cross-sectional and prospective) | Participation: moderate  Attrition: high  Belief Measurement: low  Prospective Outcome Measurement: low  Study Confounding: low  Statistical Analysis & Reporting: low |  |
| Switzerland Musculoskeletal Health Survey | | | | | |
| Elfering A, 2009, Switzerland, 264 | Population-based survey of adults from German-speaking area of Switzerland.  Current BP. | FABQ-PQ  FABQ-Work  BBQ | Beliefs’ associations with BMI, perceived heavy workload, and sport participation (RQ2);  Beliefs as predictors of future pain intensity, frequency, recovery and perceived work impairment (RQ3, prospective) | Participation: moderate  Attrition: moderate  Belief Measurement: low  Prospective Outcome Measurement: low  Study Confounding: low  Statistical Analysis & Reporting: low | |
| Elfering A, 2015, Switzerland, 2507 | As described above in Elfering *et al.*, 2009.  Presence or absence of BP within both of the 4-week periods preceding two of the surveys. | BBQ | Prevalence (RQ1);  Beliefs as predictors of pain intensity (RQ3, cross-sectional and prospective). | Participation: moderate  Attrition: moderate  Belief Measurement: low  Prospective Outcome Measurement: low  Study Confounding: low  Statistical Analysis & Reporting: low | |
| Mannion AF, 2009, Switzerland, 670 | As described above in Elfering *et al.*, 2009.  Current BP. | FABQ-PQ  FABQ-Work  BBQ | Beliefs’ associations with perceived reduction in work productivity and *n* days missed work due to BP (RQ3, cross-sectional) | Participation: moderate  Attrition: n/a  Belief Measurement: low  Prospective Outcome Measurement: n/a  Study Confounding: low  Statistical Analysis & Reporting: low | |
| Mannion AF, 2013, Switzerland, 1071 | As described above in Elfering *et al.*, 2009.  Current BP. | FABQ-PQ  FABQ-Work  BBQ | Prevalence (RQ1);  Beliefs’ associations with gender, age, employment status, income, *n* individuals <18 in household, education level, general and mental health (RQ2); Beliefs’ associations with previous healthcare seeking (RQ3, cross-sectional) | Participation: moderate  Attrition: n/a  Belief Measurement: low  Prospective Outcome Measurement: n/a  Study Confounding: low  Statistical Analysis & Reporting: low | |
| Standalone Publications | | | | | |
| Beales D, 2015, Australia, 958 | Random sample of residents of Busselton, Western Australia who were born 1946-1964 and listed on the electoral roll.  Mixed BP. | BBQ | Prevalence (RQ1);  BBQ associations with gender, age, employment status, income, smoking status, BMI, mental health (SF-12 MCS), recency of BP (RQ2); BBQ associations with ODI score; 'interferes with normal activities'; 'interferes with physical activities'; 'usually causes work absence'; 'usually use medication for BP'; 'usually seek professional care for BP' (RQ3, cross-sectional) | Participation: low  Attrition: n/a  Belief Measurement: low  Prospective Outcome Measurement: n/a  Study Confounding: low  Statistical Analysis & Reporting: low | |
| Bowey-Morris J, 2011, UK, 1023 | Stratified random sample of Isle of Jersey residents aged ≥16, stratified by island parish (district).  Mixed BP. | BBQ | Prevalence (RQ1);  BBQ association with gender, employment status, education level, general health (5 point scale), impact on daily activities (5 point scale), work absence (ever), accepts BP-related work absence (RQ2) | Participation: low  Attrition: n/a  Belief Measurement: low  Prospective Outcome Measurement: n/a  Study Confounding: low  Statistical Analysis & Reporting: low | |
| Briggs AM, 2010, Australia, 56 | Random sample of individuals identified within the Joondalup telephone directory.  Chronic BP. | FABQ-PA  FABQ-Work  BBQ  CSQ | Correlations (independent and dependent variables not specified) between beliefs and disability (RQ2). | Participation: moderate  Attrition: n/a  Belief Measurement: low  Prospective Outcome Measurement: n/a  Study Confounding: moderate  Statistical Analysis & Reporting: moderate | |
| Darlow B, 2014, New Zealand, 602 | Random sample of adults listed on electoral roll, proportionally sampled based on Māori/non-Māori descent.  Mixed BP. | Study-specific items about: consequences, activity, rest, medical care, diagnostic imaging, prognosis, psychological influences on pain/recovery, vulnerability, pain and injury relationship, understanding the experience of BP | Prevalence (RQ1) | Participation: low  Attrition: n/a  Belief Measurement: low  Prospective Outcome Measurement: n/a  Study Confounding: n/a  Statistical Analysis & Reporting: n/a | |
| Gross DP, 2010, Canada, 5360 | Random sample of individuals listed on province telephone databases, stratified by age, gender and rural/urban residence.  Mixed BP. | BBQ  Study-specific items about: rest, activity, medicine, prognosis. | Prevalence (RQ1) | Participation: moderate  Attrition: n/a  Belief Measurement: low  Prospective Outcome Measurement: n/a  Study Confounding: n/a  Statistical Analysis & Reporting: n/a | |
| Kovacs F, 2011, Spain, 497 | All schools in Majorca, Spain were stratified based on setting, school size and school ownership. A stratified random sample of schools was then selected for participation.  Mixed BP. | Study-specific items about activity. | Prevalence (RQ1) | Participation: low  Attrition: n/a  Belief Measurement: low  Prospective Outcome Measurement: n/a  Study Confounding: n/a  Statistical Analysis & Reporting: n/a | |
| Lindal E, 1989, Sweden, 54 | Random sample of Malmö, Sweden residents born between January 1913 and December 1962, stratified by month and year of birth and gender.  Current BP. | Study-specific items about cause and prognosis. | Prevalence (RQ1) | Participation: low  Attrition: n/a  Belief Measurement: moderate  Prospective Outcome Measurement: n/a  Study Confounding: n/a  Statistical Analysis & Reporting: n/a | |
| Szpalski M, 1995, Belgium, 2660 | Random sample identified using an annually-updated population reference, stratified by gender, age, social class and residence.  Previous history of BP. | Study-specific item about prognosis. | Belief’s association with previous healthcare seeking, bed rest, use of medicine, X-ray and surgery (RQ3, cross-sectional) | Participation: low  Attrition: n/a  Belief Measurement: unclear  Prospective Outcome Measurement: n/a  Study Confounding: moderate  Statistical Analysis & Reporting: low | |
| Urquhart DM, 2008, Australia, 506 | Women identified through a community-based research database which had been set up through random sampling of the Victorian electoral roll.  Mixed BP. | BBQ | Prevalence (RQ1);  BBQ associations with pain intensity and CPG disability (RQ3, cross-sectional) | Participation: low  Attrition: n/a  Belief Measurement: low  Prospective Outcome Measurement: n/a  Study Confounding: low  Statistical Analysis & Reporting: low | |
| Vidal J, 2013, Spain, 137 | All schools in Majorca, Spain stratified based on setting, school size and school ownership. One school per strata was randomly selected and two schools from the two largest strata were then selected.  Mixed BP. | Study-specific item about cause. | Prevalence (RQ1) | Participation: low  Attrition: n/a  Belief Measurement: low  Prospective Outcome Measurement: n/a  Study Confounding: n/a  Statistical Analysis & Reporting: n/a | |
| Waddell G, 2007, UK, approximately 2000 | Individuals were participants in a Scottish Consumer Omnibus survey.  Mixed BP. | Study-specific items about rest and activity. | Prevalence (RQ1) | Participation: high  Attrition: n/a  Belief Measurement: unclear  Prospective Outcome Measurement: n/a  Study Confounding: n/a  Statistical Analysis & Reporting: n/a | |
| Walker BF, 2003, Australia, 1228 | Random sample of adults on the Australian electoral roll, stratified by age and gender.  Experience of BP within preceding six months. | Study-specific item about consequences. | Belief’s association with previous healthcare seeking (RQ3, cross-sectional) | Participation: low  Attrition: n/a  Belief Measurement: unclear  Prospective Outcome Measurement: n/a  Study Confounding: low  Statistical Analysis & Reporting: low | |
| **Abbreviations:** | BBQ – Back Beliefs Questionnaire;  BP – back pain;  CSQ – Coping Skills Questionnaire;  CPG – Chronic Pain Grade;  FABQ-PA/Work – Fear-Avoidance Beliefs Questionnaire-Physical Activity/Work;  (m)FABQ-PA – (modified) Fear-Avoidance Beliefs Questionnaire-Physical Activity;  ODI – Oswestry Disability Index;  PCS – Pain Catastrophizing Scale;  RQ – Research Question  SF-36 – 36 item Short Form Health Survey  TSK-G/SV – Tampa Scale of Kinesiophobia-General Population/Short Form | | | | |
|  | | |  | | |

| Supporting Information Appendix S3. *Description of questionnaire instruments measuring beliefs about back pain* | | |
| --- | --- | --- |
| *Questionnaire* | *Description* | *Sample items* |
| *Construct: Beliefs about the consequences resulting from an episode of back pain* | | |
| Back Beliefs Questionnaire (BBQ) (Symonds et al., 1996) | Measure of the perceived inevitability of future consequences resulting from back pain.  This measure consists of 14 statements (including 5 distractor items) that individuals are typically asked to rate their agreement with on a 1-5 Likert scale where a higher than neutral score represents disagreement with beliefs about the possible negative consequences resulting from back pain. | Back trouble will eventually stop you from working. |
| *Construct: Beliefs about fear of pain, movement, and consequent activity avoidance* | | |
| Fear-Avoidance Beliefs Questionnaire – Physical Activity and Work subscales (FABQ-PA, FABQ-Work) (Waddell et al., 1993) | Measure of an individual’s fear of pain and beliefs about avoidance of activities. The measure includes two distinct scales to assess fear-avoidance beliefs about both physical and work-related activities. In its original form, the statements are framed to be about “my pain” and is therefore only appropriate, and therefore included in this review, for individuals currently experiencing back pain.  Within the articles in this review, the physical activity scale consisted of four items and the work scale consisted of 11 items. Items are typically scored on a 0-6 Likert scale where a higher than neutral score represents stronger agreement with fear-avoidance beliefs. | I should not do physical activities which might make my pain worse.  My work might harm my back. |
| Fear-Avoidance Beliefs Questionnaire (modified) **–** Physical Activity subscale (mFABQ-PA) (Linton et al., 2000a) | This modified version of the FABQ-PA rephrased statements so that individuals without current back pain could interpret and respond to the statements appropriately. The statements were preceded by the following introduction: “For research purposes, we would like you to answer these questions concerning the relationship between neck or back pain [spinal pain] and activities.” | Such pain is caused by physical activity.  Physical activity might be harmful. |
| Tampa Scale of Kinesiophobia (modified) **–** General Population (TSK-G) (Picavet et al., 2002) | Measure of fear of movement and (re)injury. In its original form the questionnaire is only appropriate for individuals reporting current musculoskeletal pain. This modified version reframed statements so that individuals without current back pain could complete the questionnaire.  Responses to 17 items were assessed using a 1-4 Likert scale, with a higher than neutral score indicating stronger agreement with beliefs about fear of movement and (re)injury. | It’s really not safe for a person with low back pain to be physically active.  Simply being careful not to make unnecessary movements is the safest thing I can do to prevent back pain. |
| Tampa Scale of Kinesiophobia (modified**) –** Short Version (TSK-SV) (Leeuw et al., 2007) | This shortened version of the TSK was developed by selecting a subset of eight (out of 17 items) from the original questionnaire. This version of the questionnaire asks about general pain beliefs and is therefore only appropriate for this review when individuals reported current back pain. | If I were to try to overcome it, my pain would increase.  My body is telling me I have something dangerously wrong. |
| *Construct: Catastrophising* | | |
| Pain Catastrophising Scale (PCS) (Sullivan et al., 1995) | Measure of three dimensions of catastrophising: rumination, magnification and helplessness. This measure is not specifically about back pain and is therefore only included in the review when the sample represents individuals with current back pain.  As reported within the review, responses to 13 items were assessed using a 1-5 Likert scale, with a higher than neutral score indicating stronger agreement with catastrophising beliefs. | When I am in pain…  …There is nothing I can do to reduce the pain.  …It’s terrible and I think it’s never going to get any better. |
| Coping Skills Questionnaire – Catastrophising subscale (CSQ-Catastrophising) (Rosenstiel and Keefe, 1983). | Subscale assessing one’s tendency to have catastrophising cognitions while experiencing back pain. This measure is not specifically about back pain and is therefore only included in the review when the sample represents individuals with current back pain.  This subscale consists of six items which are typically rated on a 7-point Likert scale, where a higher than neutral score indicates stronger agreement with catastrophising beliefs. However, the study reported in this review used a 5-point scale. | I worry all the time about whether it [back pain] will end. |

| Supporting Information Appendix S4. *Characterisation of individual belief statements and prevalence of agreement with each statement* (*ranked within each thematic grouping according to prevalence of agreement*) | |
| --- | --- |
| Belief Statement | *Proportion of Agreement*  *(95% CI)* |
| *Beliefs about consequences of back pain* | |
| Statement about having fear that low back pain could impair future work capacity. ^a^ | - |
| Having back pain makes it difficult to enjoy life. | 94.1% (91.9-95.9)^b^ |
| *Beliefs about the risks or benefits of activity while experiencing back pain* | |
| If you compete in any sport, you must follow your trainer's instructions in order to avoid hurting your back. | 94% (91.9 - 96.1)^c^ |
| Lifting without bending your knees is not safe for your back. | 93.5% (91.2 - 95.3)^b^ |
| If your back hurts, you should avoid bed rest and keep as physically active as possible. | 91% (88.8 - 93.5)^c^ |
| If you have back pain you should try to stay active. | 40% (37.9 - 42.1)^g^; 55.5% (52.5 - 58.5) to 62.8% (59.9 - 65.7)^h^; 80.0% (76.6 - 83.1)^b^ |
| Physical activity and sport is bad for your back. | 10.9% (8.2 - 13.6)^c^ |
| The more you exercise and practice sport, the healthier your back. | 74% (70.1 - 77.9)^c^ |
| Bending your back is good for it. | 59.3% (55.2 - 63.3)^b^ |
| If an activity or movement causes back pain, you should avoid it in the future. | 58.9% (54.8 - 32.9)^b^ |
| When you have back pain the risks of vigorous exercise outweigh the benefits. | 55.1% (51.0 - 59.2)^b^ |
| The longer you remain seated, the healthier your back. | 51.8% (47.4 - 56.2)^c^ |
| Sitting is bad for your back. | 42.7% (38.6 - 46.8)^b^ |
| One recovers faster from back pain if one continues at work, or return as soon as possible. | 31.2% (27.1 - 35.3) to  40.3% (36.0 - 44.6)^d^ |
| If you have back pain, you should avoid exercise. | 24.9% (21.4 - 28.6)^b^ |
| *Beliefs about the important of rest during an episode of back pain* | |
| If your back hurts, you should take it easy until the pain goes away. | 70% (65.4 - 73.0)^b^;  25.5% (22.7 - 28.3)^e^;  21% (18.5 - 23.5)^f^ |
| If you have back pain you should rest until it gets better. | 55% (58.2 - 57.2)^g^;  44.4% (41.4 - 47.4) to 47.1% (44.1 - 50.1)^h^ |
| Low back pain should have rest and tranquillity until recovery. | 41.9% (37.6 - 46.2) to 45.7% (41.4 - 50.0)^d^ |
| Bed rest is the mainstay of therapy. | 12% (10.0 - 14.0)^e,f^ |
| *Beliefs about the role of medicine in treating back pain* | |
| Simple painkillers are usually enough to control most back pain. | 24.9% (22.3-27.5) to 27.3% (24.7-29.9)^h^ |
| *Beliefs about the necessity of medical care or treatment* | |
| It is important to see a health professional when you have back pain. | 84.8% (81.7 - 87.6)^b^ |
| If you have a slipped disc, you must have surgery. | 41.2% (37.8 - 44.6)^f^;  29% (26.2 - 31.8)^e^ |
| *Beliefs about the relevance of diagnostic imaging or receiving a diagnosis for back pain* | |
| X-ray and newer imaging tests can always identify the cause of pain. | 43% (40 - 46)^e^ ; 50.9% (47.8 - 54.0)^f^ |
| Modern X-rays will usually identify the cause of pain. | 45% (41.9-48.1) to 54% (49.6-58.4)^l^ |
| Everyone with back pain should have a spine X-ray. | 47% (42.6-51.4) to 57% (52.7 - 61.3)^l^; 50.0% (46.9 - 53.1)^e^; 58.1% (55.1 - 61.1)^f^ |
| To effectively treat back pain, you need to know exactly what is wrong. | 86.2% (83.2-88.9)^b^ |
| *Beliefs about causal attributions* | |
| Most back pain is caused by injuries or heavy lifting. | 49% (45.8 - 52.2)^f^;  49% (45.9 - 52.1)^e^ |
| Statement about whether individuals thought they knew the cause of their [current] pain. | 44% (30.8 - 57.2)^i^ |
| Belief that backpack weight does not affect the back. | 13.9% (8.1 - 19.7)^j^ |
| *Beliefs about back pain’s prognosis and the natural history of back pain* | |
| Most back pain settles quickly, at least enough to get on with normal activities. | 63.9% (59.9 - 67.8)^b^ |
| There is a high chance that back pain will not resolve. | 43.5% (39.4 - 47.5)^b^ |
| Statement about whether individual believed their low back pain would be a lifelong problem. ^k^ | - |
| Statement about whether individual believed pain would last forever. | 37% (24.1 - 49.9)^i^ |
| Most back pain settles quickly and you can get on with normal activities such as going to work. | 25% (22.4 - 27.6) to 28.6% (25.9 - 31.3)^h^ |
| Back pain recovers best by itself. | 20.1% (16.6 - 23.6) to 21.9% (19.3 - 24.5)^d^ |
| Once you have a back problem, there is not a lot you can do about it. | 15.9% (13.1 - 19.1)^b^ |
| *Beliefs about psychological influences on recovery* | |
| Stress in your life (financial, work, relationship) can make back pain worse. | 63.6% (59.6 - 67.5)^b^ |
| Thoughts and feelings can influence the intensity of back pain. | 58.2% (54.1 - 62.2)^b^ |
| Focusing on things other than the back helps you to recover from back pain. | 55.5% (51.4 - 59.6)^b^ |
| Worrying about your back can delay recovery from back pain. | 54.3% (50.2 - 58.3)^b^ |
| Expecting your back pain to get better helps you to recover from back pain. | 52.4% (48.3 - 56.5)^b^ |
| *Beliefs about the vulnerability of the back* | |
| Good posture is important to protect your back. | 98.7% (97.4 - 99.4)^b^ |
| It is important to have strong muscles to support your back. | 96.5% (94.7 - 97.8)^b^ |
| You could injure your back if you are not careful. | 94.9% (92.8 - 96.6)^b^ |
| It is easy to injure your back. | 89.3% (86.5 - 91.6)^b^ |
| Your back is well designed for the way you use it in daily life. | 77.8% (74.2 - 81.1)^b^ |
| Your back is one of the strongest parts of your body. | 76.0% (72.4 - 79.4)^b^ |
| If you overuse your back it will wear out. | 51.8% (47.8 - 55.9)^b^ |
| Once you have back pain there is always a weakness. | 51.8% (47.7 - 55.8)^b^ |
| *Beliefs about the relationship between pain and injury* | |
| If you ignore back pain, you may cause damage to the back. | 89.3% (86.5-91.7)^b^ |
| You can injure your back and only become aware of the injury some time later. | 83.9% (80.8-86.8)^b^ |
| A twinge in your back can be the first sign of a serious injury. | 63.9% (59.9-67.7)^b^ |
| Back pain means that you have injured your back. | 56.5% (52.4-60.5)^b^ |
| When you have back pain, you can do things which increase your pain without harming the back. | 39.4% (35.5-43.5)^b^ |
| *Beliefs about understanding back pain* | |
| It is hard to understand what back pain is like if you have never had it. | 92.9% (90.5 - 94.8)^b^ |
| It is worse to have pain in your back than your arms or legs. | 72.1% (68.3 - 75.7)^b^ |
| Back pain is usually disabling. | 22.5% (19.8 - 25.2)^f^;  19% (16.6 - 21.4)^e^ |
| **Study Key:**   1. Walker et al., 2004; association between agreement with belief and previous healthcare-seeking investigated | |
| 1. Darlow et al., 2014; New Zealand; ≥18 years old; 2012 data collection | |
| 1. Kovacs et al., 2011; Spain; 8 year old schoolchildren; 2008 data collection | |
| 1. Werner et al., 2008; Norway; ≥15 years old; 2002-2005 data collection (multiple time-points) | |
| 1. Ihlebæk and Eriksen, 2005; Norway; ≥15 years old; 2003 data collection | |
| 1. Ihlebæk and Eriksen, 2003; Norway; ≥15 years old; 2001 data collection | |
|  | |
| 1. Waddell et al., 2007; Scotland, UK; 2000 data collection | |
| 1. Gross et al., 2010; Canada; 2005-2008 data collection (multiple time-points) | |
| 1. Líndal and Udèn, 1989; Sweden; adults; unknown year of data collection | |
| 1. Vidal et al., 2013; Spain; 10-12 year old schoolchildren; 2007 data collection | |
| 1. Szpalski et al., 1995; association between agreement with belief and previous illness behaviours investigated | |
| 1. Werner et al., 2009; Norway; ≥15 years old; 2002-2005 data collection (multiple time-points) | |
|  | |

| Supporting Information Appendix S5. *Number of studies contributing to each review question using each of the described beliefs* | | | |
| --- | --- | --- | --- |
|  | Review question | | |
| *Belief about back pain instrument / unique item theme* | *N studies reporting prevalence of a belief*  *(% of 16 studies)* | *N studies reporting an association between a belief and some factor (where belief represents hypothesised dependent variable), or correlate of a sociodemographic factor*  *(% of 5 studies)* | *N studies reporting an association between a belief and some factor (where belief represents hypothesised independent variable)*  *(% of 8 studies)* |
| *Back pain belief instrument* | | | |
| BBQ | 6 (38%) | 3 (60%) | 3 (38%) |
| FABQ-PA | 1 (6%) | 1 (20%) | 1 (13%) |
| (m)FABQ-PA | 2 (13%) | 0 | 2 (25%) |
| TSK-G | 1 (6%) | 0 | 1 (13%) |
| TSK-SV | 1 (6%) | 0 | 1 (13%) |
| FABQ-Work | 1 (6%) | 1 (20%) | 1 (13%) |
| PCS | 0 | 0 | 1 (13%) |
| *Back pain belief item theme* | | | |
| Consequences | 1 (6%) | 0 | 1 (13%) |
| Risks or benefits of activity | 5 (31%) | 1 (20%) | 0 |
| Importance of rest | 5 (31%) | 2 (40%) | 0 |
| Role of medicine | 1 (6%) | 0 | 0 |
| Necessity of medical care / treatment | 3 (19%) | 2 (40%) | 0 |
| Relevance of diagnostic imaging / diagnosis | 3 (19%) | 2 (40%) | 0 |
| Causal attributions | 3 (19%) | 1 (20%) | 0 |
| Prognosis and natural history | 4 (25%) | 1 (20%) | 1 (13%) |
| Psychological influences on recovery | 1 (6%) | 0 | 0 |
| Understanding the back (vulnerability) | 1 (6%) | 0 | 0 |
| Understanding back pain (pain/injury relationship) | 1 (6%) | 0 | 0 |
| Understanding what back pain is like | 2 (13%) | 1 (20%) | 0 |

Supporting Information Appendix S6. Factors associated with beliefs about back pain: results from cross-sectional studies where beliefs were hypothesised as the dependent variable, or investigated as a correlate of a sociodemographic or general health-related factor

| **Article** | **Sample** (country, *n*, description) | **Associated Factor** | **Belief** | **Statistical Analysis** | **Results** |
| --- | --- | --- | --- | --- | --- |
| Mannion et al., 2013 | Switzerland, 1071, current BP (Elfering *et al.*, 2015 subset) | Gender, age, employment, income, *n* individuals <18 in household, education, general health (1-7 scale), mental well-being (EuroQol) | BBQ; FABQ-PA and Work | Pearson correlation | All beliefs correlated with being older, having fewer individuals <18 in household, having completed less education, poorer general health and mental well-being (*p*<0.05). Males more likely to agree with BBQ and FABQ-Work items (*r*=-0.07, 0.08 respectively; *p*<0.05). BBQ and FABQ-PA correlated with being less employed (*r*=-0.12, -0.11 respectively; *p*<0.001); BBQ and FABQ-Work correlated with lower income (*r*=-0.08, -0.15 respectively; *p*<0.01). |
| Elfering et al., 2009 | Switzerland, 264, current BP (Elfering *et al.*, 2015 subset) | BMI, sport participation, perceived heavy workload | BBQ; FABQ-PA and Work | Pearson correlation | All beliefs significantly correlated with higher BMI and lower levels of participation in sports (*p*<0.05). |
| Bowey-Morris et al., 2011 | UK, 1023, mixed BP | Gender, employment, education, general health (5 pt scale), impact on daily activities (5 pt scale), work absence (ever), accepts BP-related work absence | BBQ | Pearson correlation, multiple regression | BBQ associated with having completed less education (β =0.30, *p*<0.001), having more daily limitations (β =0.18, *p*<0.01), accepting BP-related work absence (β =-0.21, *p*<0.01), and having missed work due to BP (β =-0.17, *p*<0.05) (BP group). BBQ scores also associated with having completed less education (β =0.09, *p*<0.001) accepting BP-related work absence (β =0.25, *p*<0.01) and poorer general health (β =0.12, *p*<0.05) (no pain group). |
| Beales et al., 2015 | Australia, 958, mixed BP | Gender, age, employment, income, smoking, BMI, mental health (SF-12 MCS), recency of BP | BBQ | Univariate regression (unadjusted and adjusted for other sociodemo-graphic variables) | Within adjusted analyses, BBQ scores associated with being on sick-leave/disability (B=3.34, *p*<0.01), lower income (B=0.49, *p*<0.01), poorer mental health (B=1.06, *p*<0.01) and more recent experience of BP (B=1.39, *p*<0.01). Within unadjusted analyses, BBQ scores associated with being older (B=-0.70, p<0.001) and by being a smoker (B=-1.44, *p*<0.05). |
| Ihlebæk et al., 2003 | Norway, 1015, mixed BP | Age (used as determinant of 1 item), education level (used as determinant of seven items) | Items about rest, treatment, imaging, cause, under-standing BP experience | Univariate logistic regression adjusted for sociodemo-graphic variables | More agreement with all items associated with having completed less education (*p*<0.05). Agreement with a belief about back pain usually being disabling was associated with being younger (OR: 0.48, 95% CI: 0.30-0.78). |
| Werner et al., 2008 | Norway, 2507, mixed BP | County of residence | Items about activity, rest, treatment, imaging, prognosis | Univariate logistic regression adjusted for sociodemo-graphic variables and BP | Residents within two specified counties more likely to agree with a statement about the need for surgery (OR: 1.6, 95% CI: 1.2-2.2). |

Supporting Information Appendix S7. Correlates of beliefs about back pain: relationships between beliefs and factors other than sociodemographic/health variables (where the independent and dependent variables were not specified)

| **Article** | **Sample** (country, *n*, description) | **Belief** | **Correlate** | **Statistical Analysis** | **Results** |
| --- | --- | --- | --- | --- | --- |
| Mannion et al., 2013 | Switzerland, 1071, current BP (Elfering *et al.* 2015 subset) | BBQ; FABQ-PA and -Work | BP episode frequency, BP intensity, limitations in activities of daily living, BBQ, FABQ-PA, FABQ-Work | Pearson correlation | All beliefs correlated with back pain frequency (*r*=0.20-0.31, *p*<0.001), intensity (*r*=0.16-0.30, *p*<0.001), and limitations in activities of daily living (*r*=0.42-0.53, p<0.001). All beliefs were also significantly correlated with each other (*r*=0.42-0.51, *p*<0.001). |
| Elfering et al., 2015 | Switzerland, 2507, mixed BP | BBQ | Recency of BP experience | Pearson correlation | BBQ correlated with having more recent/current BP (*r*=0.08*, p*<0.05). |
| Briggs et al., 2010 | Australia, 56, current chronic BP | BBQ, FABQ-PA and Work, CSQ | Oswestry Disability Index (ODI) | Pearson correlation | BBQ, FABQ-PA and CSQ significantly correlated with ODI scores (*r*=0.17, 0.30, 0.15 respectively; *p*<0.01). |
| Linton et al., 2000a | Sweden, 449, no spinal pain in preceding year | mFABQ | Test of physical functioning, catastrophising (PCS) | Spearman correlation | mFABQ significantly correlated with catastrophising (*rho*=0.23, *p*<0.05) but not a test of physical functioning. |
